# Oral Plexiform Neurofibroma Involving the Buccal Mucosa: A Case Report

**DOI:** 10.7759/cureus.70514

**Published:** 2024-09-30

**Authors:** N. Fazulunnisa Begum, Karthikeyan Ramalingam, Pratibha Ramani, Arun M

**Affiliations:** 1 Oral Pathology and Microbiology, Saveetha Dental College and Hospitals, Saveetha Institute of Medical and Technical Sciences, Saveetha University, Chennai, IND; 2 Oral and Maxillofacial Surgery, Saveetha Dental College and Hospitals, Saveetha Institute of Medical and Technical Sciences, Saveetha University, Chennai, IND

**Keywords:** excision, immunohistochemistry, neurofibroma, neurofibromatosis, oral mucosal lesions, oral pathology, oral plexiform neurofibroma, plexiform neurofibroma, recurrence, surgery

## Abstract

Oral plexiform neurofibroma is a benign tumor of the peripheral nerves. It shows multiple nerve bundles with a "plexus" appearance, creating a "bag of worms" texture. They carry higher recurrences and an increased risk of malignant transformation. We present a case of a young male with an oral plexiform neurofibroma in the buccal mucosa. The patient was advised to do a full-body examination to rule out neurofibromatosis. Although surgical excision is the mainstay of treatment for oral plexiform neurofibromas, the recurrence rate could be high. The present case report aims to explore the potential mechanisms underlying recurrences even after what is considered a complete surgical removal. The involvement of critical anatomical structures may limit the extent of excision. Understanding the exact pathogenesis of recurrence is essential for improving surgical outcomes and developing more effective management.

## Introduction

Neurofibromas account for 5% of all benign soft tissue tumors. Approximately 29% of the neural lesions occur in the oral and maxillofacial regions [1-4]. The World Health Organization classification 2017 defines it as a benign peripheral nerve sheath tumor composed of a mixture of palisade cells, neuropericytes, fibroblast mast cells, and residual interspersed myelinated and unmyelinated axons in a myxoid collagenous extracellular matrix [2,3]. Oral neurofibroma was reported by Bruce in 1954, and it occurs in two forms: a solitary tumor or as one of the components of neurofibromatosis. Multiple oral neurofibromas are always noted in approximately 72% of neurofibromatosis type I (NF1) patients. Sporadic (non-NF1-associated) oral neurofibromas occur without systemic involvement [5,6].

Although anatomical factors may influence the clinical presentation of neurofibroma, the fundamental molecular mechanisms are the same. Pathogenesis involves *NF1* gene mutation leading to the loss of neurofibromin, dysregulation of the rat sarcoma virus (*RAS*),mitogen-activated protein kinase (*MAPK*) pathways, phosphatidylinositol-3 kinase (*PI3K*), protein kinase B (*AKT*), and mammalian target of rapamycin (*mTOR*) pathway, which leads to uncontrolled cell proliferation. Schwann cells are prime drivers of tumor development, and interaction from fibroblasts, mast cells, and perineural cells and axons contributes to the tumor microenvironment [6]. Plexiform neurofibroma has a higher recurrence and an increased risk of malignant transformation [4,5]. We present a case of a young male with plexiform neurofibroma in the buccal mucosa.

## Case presentation

An 18-year-old male patient reported to the Saveetha Dental College and Hospitals with a complaint of pain in the right lower back teeth region for the past few days. Past dental history revealed extraction of lower molars a few years ago. The patient was healthy on physical examination without any major systemic illnesses. There were no obvious extraoral findings.

**Figure 1:**
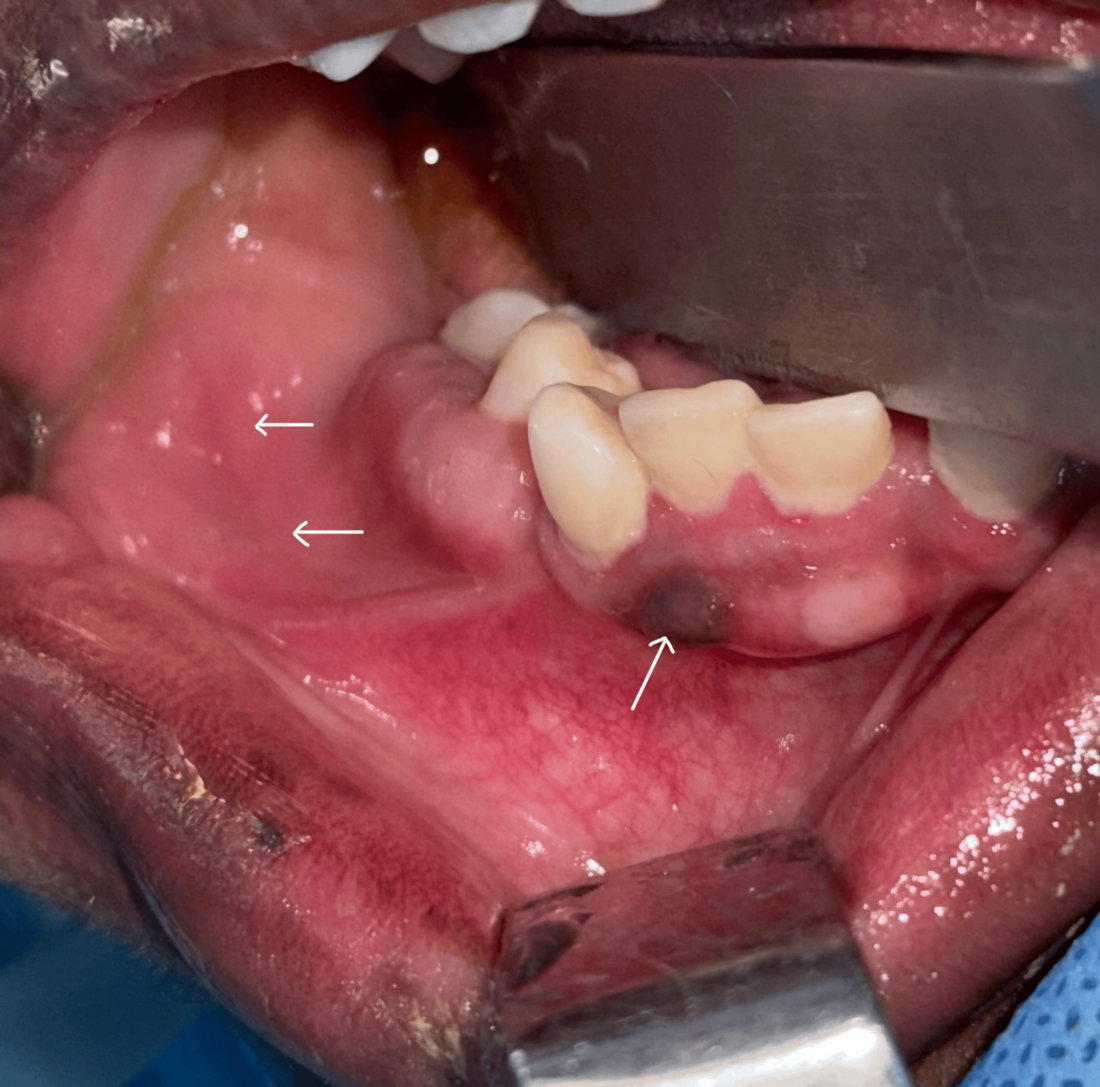
Intraoral image showing mucosal involvement and hyperpigmentation of the gingiva The lesion is shown on the buccal mucosa of the premolar region. Pigmentation is seen on the attached gingiva of the mandibular lateral incisor region.

On intraoral examination, a non-pedunculated, non-ulcerated, non-tender, non-inflamed soft tissue nodule of approximately 2 × 1.5 cm was seen in the right buccal mucosa. Unilateral jaw malformation with tooth migration of the mandibular right canine and right premolars was noted. Overall, infra-occlusion was also seen. Focal hyperpigmentation on the attached gingiva was also evident in the mandibular anterior gingiva (Figure 1).

The radiological examination was non-contributory, and the serological findings were within normal limits. Based on the clinical examination, a provisional diagnosis was given as a benign soft tissue tumor, possibly neurofibroma/lymphangioma/hemangioma. An excisional biopsy was performed under local anesthesia, and the excised tissue sample was submitted to the oral pathology department for further processing.

**Figure 2:**
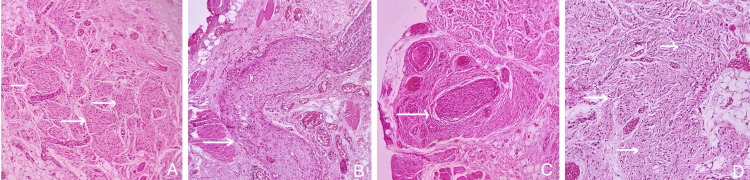
Photomicrographs (A) Shows nerve elements resembling a bag of worms appearance (H&E, 10×); (B) shows nerve bundles (H&E, 10×); (C) shows large nerve bundles (H&E, 10×); (D) shows neural elements resembling a carrot-shredded appearance (H&E, 10×). H&E: hematoxylin and eosin.

Histopathological sections showed a dense mature connective tissue stroma with the proliferation of multiple nerve bundles in a multi-nodular appearance, imparting a bag of worm/ropy gross appearance, surrounded by a thick sheath, suggestive of perineurium in a few areas. There was evidence of numerous well-formed medium-sized blood vessels, mast cells, and nerve tissue along with the skeletal muscles (Figure 2). Abundant adipocytic tissue, hemorrhage, minor mucous salivary gland acini, and mild chronic inflammatory cell infiltrate were noted.

**Figure 3:**
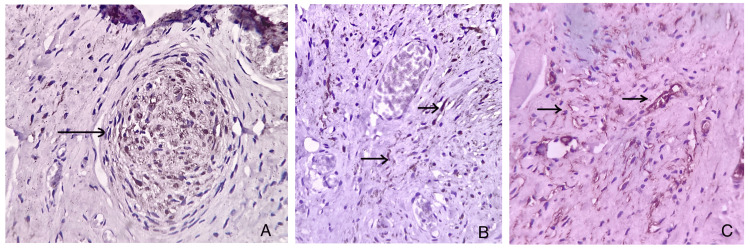
Photomicrographs (A) S-100 positivity within the nerve bundles (IHC, 40×); (B) S-100 positivity among spindle-shaped cells in the stroma and the vicinity of nerve bundles (IHC, 40×); (C) CD34 positivity observed in the stromal cells and capillaries (IHC, 40×). IHC: immunohistochemistry.

Immunohistochemistry was performed for S-100 and CD34 (Dako, Agilent, CA, USA) following the manufacturer's instructions. It revealed diffuse nuclear and cytoplasmic positivity in nerve fascicles and spindle-shaped Schwannian cell population with S-100, and diffuse cytoplasmic positivity was observed with CD34 (Figure 3).

**Figure 4:**
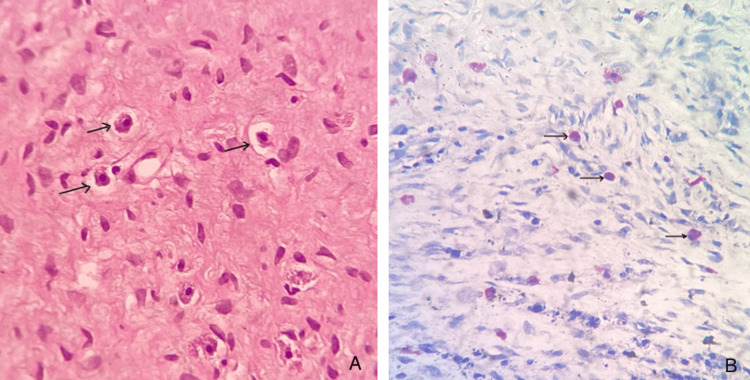
Photomicrograph (A) Mast cells seen in the connective tissue stroma as ovoid eosinophilic cells with central nuclei (H&E, 40×); (B) mast cells identified by a special stain with purple cytoplasm and blue nuclei (toluidine blue, 40×).

A special stain was performed to highlight the mast cells. Toluidine blue staining revealed the presence of mast cells (Figure 4).

Correlating clinical features and histopathological findings, the diagnosis was plexiform neurofibroma. The patient was advised for a full-body examination to rule out neurofibromatosis and be under constant follow-up.

## Discussion

The present case exhibited swellings in the oral mucosa and craniofacial manifestations, such as infra-occlusion, unilateral jaw malformation, and tooth migration. An oral plexiform neurofibroma was confirmed with characteristic histopathological findings and strong positivity for S100, CD34, and toluidine blue staining.

Neurofibromas are benign neoplasms with subtypes including cellular, ancient, plexiform, and atypical variants. They exhibit distinct DNA methylation profiles, indicating potential differences in their cellular origin. The relationship between cranio-maxillofacial malformations and neurofibromas is not fully understood, but the most severe deformities are typically linked to plexiform neurofibromas [5-8].

The updated diagnostic criteria for NF1 by NF Midwest 2021 [7] include six or more café-au-lait macules >5mm in prepubertal children, >15 mm post-pubertal individuals, freckling in armpit/groin, two or more neurofibroma or one plexiform neurofibroma, two or more Lisch nodules or two or more choroidal abnormalities, optic pathway glioma, osseous lesion such as sphenoid dysplasia, tibial dysplasia, or pseudarthrosis of a long bone, pathogenic *NF1* gene variant, parent with NF1 by the above criteria, and at least one of the two pigmentary findings (café-au-lait macules or freckling) should be bilateral [7-10]. Among the two types of NF, NF1 is more common than NF2 [11-14]. Plexiform neurofibromas are often associated with unilateral jaw malformations and tooth migration. While neurofibromas may be present during the primary dentition, they do not typically affect the development or size of permanent teeth. However, infra-occlusion of the teeth is often observed in NF1 patients [5,7]. Additionally, facial deformities that manifest in childhood tend to stabilize and show little progression in adulthood, as the growth potential of plexiform neurofibromas diminishes with age [5,8,9].

Surgical removal of plexiform neurofibromas carries a significant risk of recurrence. Possible reasons for the recurrence could be the diffuse and multinodular growth pattern and cellular characteristics. Microscopic extensions of the tumor beyond visible margins could have been missed during surgery, leading to recurrence as the residual cells proliferate. Complex perineural involvement and a conservative approach to prevent facial disfigurement and protect critical structures that make the complete excision challenging [9-13]. Oral plexiform neurofibromas, particularly in patients with NF1, have a high recurrence rate [15,16]. This could be due to their infiltrative growth along nerve sheaths and the challenges of achieving complete surgical removal. Margaret et al. stated that haploinsufficiency of the *NF1* tumor suppressor gene in the microenvironment and nerve injury may promote tumorigenesis by providing a selective advantage to tumor cells and their precursors and altering the function of non-neoplastic cells in the tumor environment. Additionally, residual mast cells, influenced by their *NF1 *genotype, may transition to wild-type mast cells, contributing to tumorigenesis in the affected area [11]. Thus, *NF1* haploinsufficiency and residual mast cells could also be presumed reasons for recurrence. Further insilico analysis and molecular studies using both the fresh and archived specimens can be performed to understand the basic and exact pathogenesis of multiple recurrences [17,18].

Although asymptomatic, multiple recurrences within a short period following wide surgical excision are a valid concern. In contrast to the present case, Suramya et al. stated that solitary neurofibromas treated by complete excision had little chance of recurrence and had no complications over the follow-up period of 12 months [14]. Moreover, multiple recurrences have been associated with an increased risk of malignant transformation [9]. The malignant transformation has been well-documented for decades, with an incidence of 3% to 5%. Malignant peripheral nerve sheath tumors (MPNSTs) often arise in association with diffuse plexiform neurofibromas with NF1, and these lesions generally carry a poor prognosis [9,13].

Zhao et al. reported a case of a 73-year-old male with multiple recurrent neurofibromas in the abdominal wall over 16 years, during which the patient underwent 13 surgical procedures. The authors emphasized that primary surgery should be planned to ensure radical resection and minimize the risk of postoperative recurrence, which could otherwise lead to malignant transformation. This may adversely affect the patient's quality of life and potentially reduce their lifespan [19].

## Conclusions

Plexiform neurofibromas represent an atypical form of NF1 with a heightened risk of recurrence following surgical intervention. Recurrent tumors carry a significant risk of malignant transformation. Despite advances in surgical techniques aimed at minimizing recurrence and preserving oral function, achieving complete excision remains a challenge. Intraoperative frozen sections can be incorporated to achieve clear surgical margins and minimize the risk of local recurrence.
